# Seroepidemiological Surveillance of Livestock Within an Endemic Focus of Leishmaniasis Caused by *Leishmania infantum*

**DOI:** 10.3390/ani15111511

**Published:** 2025-05-22

**Authors:** Joaquina Martín-Sánchez, María Ángeles Trujillos-Pérez, Andrés Torres-Llamas, Victoriano Díaz-Sáez, Francisco Morillas-Márquez, Patricia Ibáñez-De Haro, Francisca L. de Torres, Antonio Ortiz, Manuel Morales-Yuste

**Affiliations:** 1 Departamento de Parasitología, Facultad de Farmacia, Universidad de Granada, Campus Universitario de Cartuja, 18071 Granada, Spain; andrestll@ugr.es (A.T.-L.); diazsaez@ugr.es (V.D.-S.); fmorilla@ugr.es (F.M.-M.); patriciaibanez@ugr.es (P.I.-D.H.); yuste@ugr.es (M.M.-Y.); 2 Sanidad Animal, Agencia de Gestión Agraria y Pesquera, Consejería de Agricultura, Pesca, Agua y Desarrollo Rural, Junta de Andalucía, 18230 Atarfe, Spain; angeles.trujillos@juntadeandalucia.es (M.Á.T.-P.); franciscal.torres@juntadeandalucia.es (F.L.d.T.); antonio.ortiz.martinez@juntadeandalucia.es (A.O.)

**Keywords:** *Leishmania* spp., seroprevalence, sheep, goat, cattle, pig, Granada province (Spain), Southern Spain, natural regions

## Abstract

Leishmaniasis by *Leishmania infantum* has a zoonotic transmission cycle involving an increasing number of mammalian hosts, forming a cooperative network. The sand fly feeding on livestock is evidenced, but clinical confirmation regarding their infection is limited. We aimed to evaluate *Leishmania* seroprevalence in livestock to assess its impact on leishmaniasis epidemiology in an endemic area located in the Mediterranean region. A cross-sectional serological study screened livestock exposure to *L. infantum* and risk factors in Southern Spain. A total of 864 serum samples of clinically healthy sheep, goats, cattle, and pigs were examined by an indirect fluorescence antibody test, using a 1/80 cut-off titre to minimize cross-reactions. Global seroprevalence was 10.8%: 21.6% cattle, 15.4% sheep, 7.3% goats, and 0.6% pigs. Statistically significant differences in positive detection were observed among species (*p* < 0.001) and natural regions (*p* < 0.001). High positive reactions in cattle, goats, and sheep suggest livestock exposure to *Leishmania* spp. and potential asymptomatic infection. Livestock presence in biotopes could promote a dilution effect, reducing human leishmaniasis incidence. Further investigation is needed to confirm livestock roles in leishmaniasis maintenance and transmission.

## Simple Summary

The life cycle of the parasite *Leishmania infantum* involves an increasing number of mammalian hosts. *Leishmania* parasites are transmitted between these mammalians by the female of a small insect known as the sand fly. There is evidence of the sand fly feeding on the blood of livestock, but there is little clinical confirmation regarding the possible infection by *L. infantum* on these animals. Our objective was to test the presence of specific antibodies against this parasite in the blood of sheep, goats, cattle, and pigs to screen the livestock exposure to *L. infantum* and risk factors in Southern Spain. A total of 864 clinically healthy sheep, goat, cattle, and pig samples were examined by indirect fluorescence antibody test. The survey revealed that 10.8% of the investigated animals had antibodies: 21.6% cattle, 15.4% sheep, 7.3% goats, and 0.6% pigs. A high percentage of livestock without clinical symptoms have antibodies against the parasite in their blood, which suggests they may have an asymptomatic infection. Further research is needed to confirm the role of these animals in maintenance and transmission of leishmaniasis.

## 1. Introduction

*Leishmania* parasites are transmitted between hosts during the blood-feeding process by infected female sand flies (Insecta, Diptera, Psychodidae), which act as biological vectors. *Leishmania infantum* is responsible for zoonotic leishmaniasis in the Mediterranean region, where an increasing number of mammalian species are being implicated as potential hosts, forming a cooperative transmission network around the main reservoirs, domestic dogs, and wild rabbits [1]. According to this, the vectors of *L. infantum* are highly opportunistic in their feeding behavior [2,3,4,5]. Feeding and host preferences play a key role in determining which mammals may contribute to the transmission of *L. infantum*. Numerous studies conducted in the Mediterranean leishmaniasis foci have demonstrated that *P. perniciosus*, the main *L. infantum* vector, exhibits opportunistic feeding habits, ingesting a variety of blood sources, including dogs, humans, rabbits, cats, cattle, goats, sheep, chicken, horses, donkeys, turkeys, pigs, and other animals [6,7,8,9,10]. Some of these studies established that a significant percentage of phlebotomine blood meals are sourced from livestock [7,8,10]. However, there remains a deficit in clinical evidence on the likelihood of infection in these animals, and little attention has been paid to livestock and the role they may play in the transmission of leishmaniasis. A few publications have shown positive detection rates for *L. infantum* in asymptomatic sheep, goats, and cattle from Iran, China, and Brazil, using serological and molecular methods [11,12,13,14], and at least one clinical case of leishmaniasis has been diagnosed in sheep, goats, and cows [15,16,17]. Recent publications in Europe showed that almost 1.5 and 10% of tested sheep from Southern Germany and Spain, respectively, and 17.3% of cattle from Southern Italy harbored antibodies against *L. infantum* [18,19,20]. An extensive survey into the possible role of these animals in the epidemiology and transmission dynamics of leishmaniasis in the Mediterranean areas should be planned.

The aim of the present study was to assess the occurrence of *Leishmania* antibodies in cattle (*Bos taurus*), sheep (*Ovis aries*), goats (*Capra hircus*), and pigs (*Sus scrofa domesticus*) from an endemic area for canine and human leishmaniasis. This investigation constitutes a preliminary step to verify the potential role of livestock in the epidemiology of leishmaniasis by *L. infantum*.

## 2. Materials and Methods

Study area: Granada Province (Spain) is in the southeast of the Iberian Peninsula, and it is part of the autonomous community of Andalusia (geographical coordinates of the polygon centroid: 36°15′ N 3°15′ W). It experiences a Mediterranean climate and has an average altitude of 1070 m above sea level (range: 0–3479) (https://www.juntadeandalucia.es/institutodeestadisticaycartografia/sima/provincia.htm?prov=18, accessed on 19 April 2025). The province comprises 10 natural geographic regions and has been considered endemic for leishmaniasis since 1913. Canine leishmaniasis (CanL) prevalence is globally high throughout the province, and a high prevalence of *L. infantum* has also been documented in wild rabbits (18.2 to 100%) and other animal species [21,22,23]. Differences in the annual incidence of autochthonous human leishmaniasis between the natural geographic regions have been associated with parasitic loads in the skin of wild rabbits [1,22].

Animals and biological samples: Serum samples from animals located in the province of Granada were obtained through the Monitoring and Control Program for Ovine and Caprine Brucellosis of the Andalusian government. A sample of farms is monitored on an annual basis to detect the presence of *Brucella* spp. with at least a 95% confidence level, with a target prevalence of 0.2% of establishments with sheep or goats. Farms in each region are randomly selected. On the farms, 100% of sheep and goats older than 6 months are monitored, along with cattle (100%, older than 12 months) and pigs (sampled for a target prevalence of 10% and a 95% confidence level in animals older than 6 months) living alongside them. Most farms were visited from late December 2022 to early March 2024. Blood samples were drawn by jugular venipuncture and collected in 10 mL tubes for serum separation by centrifugation. Species, sex, and age of each animal were recorded. Serum samples were stored at −20 °C until analysis.

Serological diagnosis of leishmaniasis: *Leishmania*-specific antibody detection was performed by an indirect fluorescence antibody test (IFAT), as previously described [24], using a homemade antigen. Briefly, a suspension of *L. infantum* promastigotes (2 × 10^6^ cells/mL) strain MCAN/ES/91/DP204 (zymodeme MON-1 = GR-1) was used. The antibody titre against *Leishmania* was determined by serial dilutions from livestock serum samples, with a starting dilution of 1/20 to final dilution of 1/1280 to determine the final titre. Positive sera were titrated by serial dilutions until negative results were obtained. MegaFLUO^®^ conjugate anti-goat, anti-sheep, anti-cattle, and anti-pig IgG fluorescein isothiocyanate (FITC)-labeled (MEGACOR Diagnostik, Hörbranz, Austria) were used according to manufacturer’s instructions. Since no standard cut-off for livestock exists, the cut-off titre of 1/80—commonly used in humans—was adopted. According to this, titres equal to or above 1/80 were considered positive. A canine serum sample positive for leishmaniasis was used as a positive control, while a serum sample from a dog negative for leishmaniasis was used as a negative control, both including anti-dog conjugate.

Statistical analysis: Species (bovine, sheep, goat, pig), breed, sex (female, male), and age of animals, along with the natural geographical region of the farm, were used as independent variables. The outcome variable was the positivity to IFAT. Logistic regression was the statistical method of choice to estimate the probability of occurrence of the event, since the dependent variable can only have a value of 0 (negative: ≤1/40) or 1 (positive: ≥80). Univariate logistic regression analyses were conducted using the IBM SPSS Statistics 21.0 software for Windows, with all the independent variables set against the dependent variable. All variables returning a value of *p* ≤ 0.2 in the univariate study were used to construct the multivariate model using a stepwise selection procedure. In the final multivariate model, variables with *p* ≤ 0.05 were retained.

## 3. Results

A total of 864 animals were investigated in the survey, including 14.6% males and 85.4% females (122 males, 712 females, and 30 pigs of unknown sex). Overall, 10.8% (93/864) of the animals showed antibody titres against *L. infantum* ≥ 1/80. Table 1 summarizes the antibody titres detected among the four livestock species. The highest titres (1/160 and 1/320) were found in cattle, sheep, and goats.

The positivity rate for males was 4.1% (5/122) and 12.2% (87/712) for females. A significant difference in positivity by sex was observed (*p* = 0.012; odds ratio = 3.26, CI95% 1.30–8.20).

Mean ages were 75.2 months (CI95% 68.1–82.3) for cattle, 60.4 months (CI95% 56–64.7) for sheep, and 39.3 months (CI95% 36.5–42.1) for goats. No age data were available for pigs, although all were older than 6 months. A statistically significant positive association was found between test positivity and increasing age in all species (*p* < 0.003; odds ratio = 1.009, CI95% 1.003–1.015).

The sample included 302 goats, 280 sheep, 157 pigs, and 125 bovines. Seroprevalence rates were 7.3%, 15.4%, 0.6%, and 21.6%, respectively. Statistically significant differences in positivity were observed among species (*p* < 0.001): cattle = sheep (*p* = 0.126) > goats > pigs. Differences by breeds were detected in sheep, with breed *Segureña* sheep showing the highest positivity risk (odds ratio = 3.14, CI 95% 1.44–6.83, *p* = 0.004).

At least one seropositive animal was found in each of the 10 natural regions of the province of Granada. Significant differences in positivity rates were observed among natural regions (*p* < 0.001), both overall and when controlled by species. Animals from the *Huéscar* and *Valle de Lecrín* regions showed the highest seropositivity risk.

Sex and age lose their statistical significance (*p* = 0.652 and 0.196, respectively) when all the four variables (sex, age, species, and natural region) were included in the model. An interaction between age and species was observed (*p* = 0.037), affecting only goat when compared to bovine (*p* = 0.011, odds ratio = 1.026 [CI95% 1.006–1.046]).

Table 2 and Table 3 summarize livestock exposure risk to *L. infantum* infection based on serological diagnoses and epidemiological variables (sex, age, species, and natural regions), as determined by univariate and multivariate logistic regression analyses.

## 4. Discussion

*Leishmania infantum* parasites can infect a wide range of mammalian species, which may act as vertebrate hosts within the parasite’s life cycle. Mediterranean hotspots are associated with the presence of networks of wild and domestic reservoirs at the interface of sylvatic and urbanized environments for the spread of leishmaniasis. Dogs and leporids are considered the main sources of infection in urban and wild areas, respectively [1,25,26]. While livestock play an important role in attracting and maintaining sand fly populations, their role in the epidemiological network of *L. infantum* remains unclear. Overall, few studies have focused on assessing the role of livestock in the epidemiology of the genus *Leishmania,* with most addressing sand fly feeding preferences. These surveys confirmed that cattle, small ruminants, and pigs serve as blood sources for sand flies, particularly *P. perniciosus* [3,4,5,6,7,9,10].

The presence of anti-*Leishmania* antibodies is a strong indication of exposure to infection, and the prevalence of infected dogs in *L. infantum* endemic areas is usually measured by serological techniques due to their ease of use and efficiency. IFAT is one of the most frequently used immunological methods, using 1/80 or 1/160 titre thresholds [26]. We applied IFAT to screen for the prevalence of anti-*Leishmania* antibodies in livestock from a well-known *L. infantum* endemic area. The relatively high seroprevalence observed (10.8%), especially in cattle and sheep, suggests significant *L. infantum* exposure. A subset of the seropositive animals displayed relatively strong antibody response (titres 1/320 and 1/640), reinforcing this observation. The high prevalence of *Leishmania*-seropositive animals observed among livestock in endemic regions, as outlined in this study, indicates a potential previous exposure of these animals to the parasite, with the possibility of an asymptomatic infection occurring. However, parasitemia was not a subject of our investigation, and it is widely acknowledged that these immunological methods are limited by their inability to discriminate between past and present infections and by the possibility of cross-reactions with other infectious agents.

In this regard, the possibility of a cross-reaction with other pathogens that infect livestock cannot be excluded. Trypanosomes, widespread hemoflagellates commonly found in vertebrates and transmitted by arthropods, may contribute to this cross-reactivity. Non-pathogenic species have been found in a range of mammalian hosts in nature, at varying prevalence levels and causing little or no apparent negative effects in the host. Such species are commonly reported as non-pathogenic trypanosomes, a category for which the host range is notably limited [27,28,29]. *Trypanosoma theileri* is a common parasite in cattle, and tabanids are thought to be the most important vectors. *Trypanosoma melophagium*, on the other hand, is a species specific to domestic sheep and is transmitted by the sheep ked, *Melophagus ovinus* [27,29,30]. In Spain, Villa et al. (2008) [27] observed microscopically *Trypanosoma* spp. in 4/18 blood samples from cattle, representing the only reference to this parasite in cattle in the country. We have no evidence of the detection of other trypanosomes, especially those belonging to the stercoraria section, in small domestic ruminants in Spain. Calzolari et al. (2018) [30] reported the presence of *T. theileri*-like trypanosomes in sand flies from the Emilia–Romagna region of Italy. The limited pathogenic effects of these species on the host have resulted in their sporadic reporting as occasional findings during parasitological surveys not specifically focused on trypanosomes. Hence, there is a deficit of knowledge about their epidemiology in Europe [29]. The reactivity of tests based on *Leishmania* promastigotes with livestock infected with *Trypanosoma* appears to be feasible, although it remains undocumented. In wild rabbits, where the prevalence of both trypanosomatids is high, a low correlation was detected between the antibody titres obtained using the two types of antigens, *L. infantum* promastigotes and *Trypanosoma nabiasi* epimastigotes [28]. Therefore, although the *Trypanosoma* infection is possible in our environment, we believe that the threshold titre selected is high enough to be indicative of prior exposure to *L. infantum*.

Furthermore, the recent detection of *Leishmania tarentolae* in humans and dogs using serological and molecular methods in southern Italy raises concerns about the possibility of livestock exposure to this nonpathogenic species, whose ecology is largely unknown [31].

Among the few studies that have reported the occurrence of *Leishmania* spp. in livestock using molecular techniques, Lobsiger et al. (2010) [16] described a clinical case of a cow in Switzerland showing symptoms compatible with leishmaniasis. The sequencing of the cow’s cutaneous tissue sample revealed 98% sequence similarity with *Leishmania siamensis*. In addition, Bhattarai et al. (2010) [32] found *Leishmania* blood infections among apparently healthy cows and goats in Nepal, and Gao et al. (2015) [11] revealed the presence of such infections among sheep, goats, and cattle in China. Paixao-Marques et al. (2018) [13] reported the occurrence of *L. infantum* in one apparently healthy bovine from an endemic area in Brazil, diagnosed by PCR from a blood culture with subsequent sequencing for species confirmation. The first clinical case of leishmaniasis in a goat was reported in Kenya, exhibiting both visceral and cutaneous lesions, and *Leishmania aethiopica* was suspected as the causative agent [33]. A second clinical case in a goat was recently reported in Spain, showing skin lesions for several months with enlargement of lymph nodes. Anti-*Leishmania* antibodies were detected at moderate levels, and the animal exhibited complete clinical recovery following treatment with meglumine antimoniate and allopurinol [17].

Bodies morphologically compatible with the amastigote stage of *Leishmania* spp. were also detected in the pinna of a sheep from South Africa. The animal developed a swollen ear and the overlying skin thickened and encrusted. This constitutes the only reported case of cutaneous leishmaniasis in a sheep, with no antibodies being sought [15].

In pigs, data concerning infections with *Leishmania* are even more scarce. However, it has been suggested that they attract sand flies, and the presence of such animals in an area increases the risk of canine infection [34]. Moraes-Silva et al. (2006) [35] detected anti-*Leishmania* antibodies in 40% of domestic swine in a visceral leishmaniasis endemic area from Brazil and their absence in pigs from a leishmaniasis-free area. In our study, the seroprevalence detected in pigs was the lowest of all the species investigated and high antibody titres were not observed. This lower prevalence could also be partially due to the younger age of the pigs, which are usually slaughtered before they are one year old. In contrast, cattle, sheep and goats were older, particularly the cattle, and a positive association between age and exposure to *L. infantum* infection was identified in these species. This cumulative effect with age is a common finding in epidemiological studies conducted in endemic areas, both in dogs and humans. This observation indicates that prolonged residence in these areas is associated with an increased probability of exposure to the parasite through the phlebotomine sand fly vector [21,36,37].

Livestock at the greatest risk of exposure to *L. infantum* were cattle, followed by sheep, then goats, and finally pigs, which had the lowest risk. This pattern corresponds to the age distribution, from the oldest to the youngest. However, there may be differences in susceptibility to infection between species and among breeds. Martínez-Sáez et al. (2025) [20] revealed differences in serum levels of cytokines between bovine breed, thereby proposing that these variations may be indicative of different immune responses. Sheep have been used in experimental infection with *L. donovani*; one sheep out of six tested developed antibodies and showed amastigotes at the cutaneous site of inoculation for up to 28 days post-infection [38]. Humoral anti-*Leishmania* immune responses have been induced in pigs inoculated with *L. infantum,* despite the absence of parasite detection [34]. These experimental infections demonstrate that livestock respond to the inoculation with live *Leishmania* by producing antibodies, which can subsequently be detected in epidemiological studies such as this survey. However, there is no direct evidence that these exposed livestock can transmit infection to the sand fly vector.

Livestock at greatest risk for exposure to *L. infantum* was found in two natural regions where no clinical cases of human leishmaniasis have occurred recently (period 2003–2024), while the lowest risk was observed in a region that has been considered classically endemic since 1913 [1,22,36]. Bauer et al. (2024) [18] showed the exposure of sheep to *L. infantum* in a non-endemic area (Southern Germany). These results are very interesting as they suggest a greater exposure of livestock to *Leishmania* in non-endemic areas or areas with less endemicity of human leishmaniasis. Moreover, declines in the occurrence of *Leishmania* in livestock could serve as a sentinel indicator for early identification of hotspots. The presence of livestock in these biotopes could be promoting a dilution effect, which is believed to reduce the incidence of human leishmaniasis. Vector-borne pathogens are among the types of parasites most likely to be influenced by changes in biodiversity. Among these diversity-responsive parasites, negative effects (dilution) and positive effects (amplification) are possible under different circumstances [39] that may be occurring in the study area. Further investigations are required to confirm the role of these animals in maintenance and transmission of leishmaniasis.

## 5. Conclusions

In the endemic areas of Southern Spain, a high frequency of livestock with positive reactions to *L. infantum* has been observed among cattle, goats, and sheep, suggesting the possibility of asymptomatic infections. The presence of livestock could be contributing to a diversity–human disease effect. Further research is needed to confirm the role of these animals in the maintenance and transmission of leishmaniasis. This research will require the use of diagnostic tools that discriminate between possible infectious agents.

## Figures and Tables

**Table 1:** Antibody titre obtained using the indirect immunofluorescence technique (IFAT) against promastigotes of the species *L. infantum* differentiating by livestock species. Negative animals: ≤40; positive animals: ≥80.

Species and Number	Antibody Titre by IFAT							Overall
	<20	20	40	80	160	320	640	≥80
Cattle: 125	58	20	20	11	12	4	0	27/125
Sheep: 280	182	19	36	24	15	3	1	43/280
Goat: 302	231	27	22	12	8	1	1	22/302
Pig: 157	136	14	6	1	0	0	0	1/157
All: 864	607	80	84	48	35	8	2	93/864

**Table 2:** Exposure of livestock to *Leishmania* spp. based on serological analyses and risk associated with sex, age, species, breed, and natural regions according to univariate logistic regression analyses. Min = minimum; Max = maximum. Ref = used as reference. -: a figure of no interest given the lack of statistical significance (*p* > 0.05).

Variable	Category/Description	N°/Overall	Seroprevalence % (≥80/Overall)	Odds Ratio	CI95%	*p* Value
Sex	Male	122/834	4.1 (5/122)	Ref.		0.012
	Female	712/834	12.2 (87/712)	3.26	1.30–8.20	
Age (months)	53.7 (51.1–56.4)	682/864	13.2 (90/682)	1.009	1.003–1.015	0.003
	Min 6, Max 250					
Species	Bovines	125/864	21.6 (27/125)	Ref.		<0.001
	Sheep	280/864	15.4 (43/280	-	-	-
	Goats	302/864	7.3 (22/302)	0.29	0.16–0.52	<0.001
	Pigs	157/864	0.6 (1/157)	0.02	0.003–0.17	<0.001
Natural regions	Vega	75/864	14.7 (11/75)	Ref.		<0.001
	Alpujarra	116/864	5.2 (6/116)	0.32	0.11–0.90	0.031
	Valle de Lecrín	25/864	20.0 (5/25)	-	-	-
	Alhama	73/864	1.4 (1/73)	0.08	0.01–0.64	0.017
	Baza	56/864	8.9 (5/56)	-	-	-
	Costa	25/864	8.0 (2/25)	-	-	-
	Guadix	106/864	13.2 (14/106)	-	-	-
	Huéscar	72/864	23.6 (17/72)	-	-	-
	Loja	291/864	8.6 (25/291)	-	-	-
	Montes	25/864	28.0 (7/25)	-	-	-
Breed of cattle	Crossbred	86/125	19.8 (17/86)	Ref		-
	Frisona	29/125	31.0 (9/29)	-	-	-
	other	10/125	10.0 (1/10)	-	-	-
Breed of sheep	Crossbred	137/280	12.4 (17/137)	Ref		0.012
	Lojeña	81/280	11.1 (9/81)	-	-	-
	Segureña	52/280	30.8 (16/52)	3.14	1.44–6.83	0.004
	Montesina	10/280	10.0 (1/10)	-	-	-
Breed of goat	Crossbred	92/302	10.9 (10/92)	Ref		-
	Murcianogranadina	191/302	6.3 (12/191)	-	-	-
	Florida	12/302	0.0 (0/12)	-	-	-
	Other	7/302	0.0 (0/7)	-	-	-

**Table 3:** Risk of livestock exposure to *Leishmania* spp. based on serological analyses according to a multivariate logistic regression model. Ref: group used as reference. -: figure of no interest given the lack of statistical significance (*p* > 0.05).

Variable	Category	N°/864	Seroprevalence % (N°/Overall)	OR [CI95%] *p* Value	OR [CI95%] *p* Value
Species				<0.001	<0.001
	Bovines	125	21.6 (27/125)	Ref	114.6 [14.3–920.8] < 0.001
	Sheep	280	15.4 (43/280	0.40 [0.21–0.76] 0.005	46.2 [6.1–350.7] < 0.001
	Goats	302	7.3 (22/302)	0.14 [0.07–0.31] < 0.001	16.5 [2.1–130.7] 0.008
	Pigs	157	0.6 (1/157)	0.01 [0.001–0.07] < 0.001	Ref.
Natural regions				<0.001	<0.001
	Vega	75	14.7 (11/75)	Ref	-
	Alpujarra	116	5.2 (6/116)	0.26 [0.09–0.75] 0.013	0.26 [0.10–0.67] 0.005
	Valle de L	25	20.0 (5/25)	4.84 [1.30–17.98] 0.019	4.74 [1.50–14.95] 0.008
	Alhama	73	1.4 (1/73)	-	-
	Baza	56	8.9 (5/56)	-	-
	Costa	25	8.0 (2/25)	-	-
	Guadix	106	13.2 (14/106)	-	-
	Huéscar	72	23.6 (17/72)	6.50 [2.47–17.16] <0.001	6.37 [2.93–13.83] < 0.001
	Loja	291	8.6 (25/291)	-	Ref.
	Montes	25	28.0 (7/25)	-	-

## Data Availability

The data sets used are available from the corresponding author on reasonable request.
